# Serum ubiquitin C‐terminal hydrolase L1 predicts cognitive impairment in patients with acute organophosphorus pesticide poisoning

**DOI:** 10.1002/jcla.22947

**Published:** 2019-06-14

**Authors:** Li Pang, Junlan Liu, Wei Li, Yan Xia, Jihong Xing

**Affiliations:** ^1^ Department of Emergency The First Hospital of Jilin University Changchun China; ^2^ Department of Gastroenterology The First Hospital of Jilin University Changchun China

**Keywords:** acute organophosphorus pesticide poisoning, cognitive impairment, ubiquitin C‐terminal hydrolase L1

## Abstract

**Background:**

To assess the usefulness of serum C‐terminal hydrolase L1 (UCH‐L1) level as a biomarker for predicting cognitive impairment in patients with acute organophosphorus pesticide poisoning (AOPP).

**Methods:**

Two hundred and seven adult patients with AOPP were included in this study. Serum UCH‐L1 levels were assessed on admission (Day 1 postpoisoning) and on Days 3 and 7 postpoisoning. The associations between serum UCH‐L1 levels, other clinical predictors, and cognitive function evaluated on Day 30 postpoisoning were investigated.

**Results:**

On multivariate analysis, serum UCH‐L1 levels on admission (odds ratio [OR] 1.889, 95% confidence interval [CI] 1.609‐3.082, *P* = 0.002) and 24‐hour APACHE II score (OR 1.736, 95% CI 1.264‐3.272, *P* = 0.012) were independent predictors of cognitive impairment on Day 30 postpoisoning. Based on the receiver operating characteristic curve, serum UCH‐L1 levels >5.9 ng/mL on admission predicted cognitive impairment on Day 30 postpoisoning with 86.1% sensitivity and 72.5% specificity (area under the curve, 0.869; 95% CI 0.815‐0.923). On admission [8.51 (6.53‐10.22) ng/mL vs 4.25 (2.57‐6.31) ng/mL, *P* < 0.001] and Day 3 [9.31 (7.92‐10.98) ng/mL vs 3.32 (2.25‐5.13) ng/mL, *P* < 0.001] and Day 7 [4.96 (3.28‐7.26) ng/mL vs 2.27 (1.55‐3.24) ng/mL, *P* < 0.001] postpoisoning, serum UCH‐L1 concentration was significantly higher in patients that developed cognitive impairment compared to those that did not.

**Conclusion:**

This study demonstrates that serum UCH‐L1 level has potential as a novel biomarker for predicting cognitive impairment 30 days after AOPP.

## 1 INTRODUCTION

Acute organophosphorus pesticide poisoning (AOPP) is an important public health problem. AOPP is associated with substantial human morbidity and mortality because of the widespread use of organophosphate compounds.^1^ The nervous system is particularly sensitive to the effects of AOPP, which manifest as cognitive impairment, including deficits in information processing, sustained attention, memory, problem‐solving, abstraction, flexibility of thinking, and depressed mood.^2^, ^3^ Patients experiencing cognitive impairment in AOPP may have long‐lasting or irreversible cognitive and behavioral sequelae and an increased mortality rate. Thus, the identification of patients with AOPP who are at risk of cognitive sequelae could guide treatment decision‐making.

Acute organophosphorus pesticide poisoning causes irreversible inhibition of acetylcholinesterase (AchE) leading to the accumulation of acetylcholine (Ach) at synapses and muscarine, nicotine, and central nervous system symptoms. Clinically, evidence of AOPP can be confirmed by measuring a decrease in blood AchE activity, which can also be used to monitor treatment response as a prognostic indicator. However, cognitive impairment in AOPP is not always associated with decreased blood AchE activity,^4^ and evidence for a relationship between blood AchE activity and impaired neurobehavioral function is limited.^5^

Ubiquitin C‐terminal hydrolase L1 (UCH‐L1) is a neuron‐specific enzyme that is highly abundant in the brain.^6^ UCH‐L1 is a deubiquitinating enzyme that is required for normal cognitive function.^7^, ^8^ UCH‐L1 has been implicated in the pathophysiology of Parkinson's disease (PD),^9^ Alzheimer's disease (AD),^10^ Huntington's disease,^11^ and epileptic seizures.^12^ Increasingly, UCH‐L1 is recognized as a biomarker of brain injury. Circulating UCH‐L1 levels are significantly increased after acute neurological insults such as traumatic brain injury, subarachnoid hemorrhage, ischemic stroke, hypoxic‐ischemic encephalopathy, and cardiac arrest,^13^, ^14^, ^15^, ^16^, ^17^, ^18^ and altered UCH‐L1 expression is an indicator of brain injury severity and a predictor of neurological outcomes.^17^, ^19^ To the authors’ knowledge, no studies have investigated the utility of serum UCH‐L1 level as a predictor of cognitive impairment after AOPP. Therefore, this prospective study compared serum UCH‐L1 levels in patients with and without cognitive impairment following AOPP to evaluate the utility of UCH‐L1 for the prediction of cognitive impairment.

## 2 MATERIALS AND METHODS

### 2.1 Study population

Patients with AOPP admitted to the Emergency Department at the First Hospital of Jilin University between January 2016 and June 2018 were eligible for this study. Inclusion criteria were as follows: (a) diagnosis of AOPP based on a history of exposure to an organophosphorus pesticide compound and symptoms of cholinergic and muscarinic toxidromes; (b) aged ≥16 years; (c) presented to the Emergency Department within 24 hours of exposure to the organophosphate compound; and (d) no prior invasive (eg, hemoperfusion) or intravenous therapies. Exclusion criteria were as follows: (a) poisoning caused by other drugs; (b) history of neurological disease or psychiatric disorders; (c) history of severe heart, lung, liver, kidney, or hematological disease; or (d) history of cancer. A control group comprised of age‐ and sex‐matched healthy individuals with no history of exposure to an organophosphorus pesticide compound was recruited from the medical center at the First Hospital of Jilin University between January 2016 and January 2017. The study protocol was approved by the Medical Ethics Committee of the First Hospital of Jilin University (Approval #: 2015‐273). Written informed consent was obtained from study participants or relatives of unconscious patients.

All patients with AOPP were administered atropine, cholinesterase, diuretic, and anti‐inflammatory agents. All patients underwent gastric lavage, catharsis, monitoring and maintenance of life‐sustaining organs, and treatment for acid/base disturbance. Patients with severe poisoning underwent hemoperfusion once a day for at least 3 days.

### 2.2 Determination of UCH‐L1 in serum

Blood samples for assessment of serum UCH‐L1 were collected within the first 24 hours of admission to hospital (Day 1 postpoisoning) and on Days 3 and 7 postpoisoning. Samples were centrifuged for 10 minutes at 1300 *g* and stored at −80°C until analysis. Serum UCH‐L1 levels were measured using an enzyme‐linked immunosorbent assay (ELISA) kit (Proteintech Group, Inc, USA), according to the manufacturer's instructions. The lower limit of UCH‐L1 detection was 0.03 ng/mL. Each sample was assayed in duplicate, and the mean of the two measurements was used in the final analyses. Researchers performing the assays were blinded to the patients’ clinical information.

### 2.3 Data Collection and Definition of Variables

Patients’ demographic data and Acute Physiology and Chronic Health Evaluation (APACHE) II score were recorded within the first 24 hours of admission. Routine clinical laboratory tests, blood lactate levels, serum cholinesterase levels, and blood gases were recorded on admission. Serum cholinesterase activity was calculated as follows: serum cholinesterase level at admission/4500 (reference value). Time from poisoning to treatment (gastric lavage), duration of hospitalization, duration of intensive care unit (ICU) stay, and mechanical ventilation time were also recorded.

The primary endpoint of this study was cognitive function evaluated on Day 30 postpoisoning using the Mini‐Mental State Examination (MMSE) questionnaire. The MMSE evaluates orientation, registration, attention and calculation, recall, and language for a maximum score of 30. A score of ≤23 indicates cognitive impairment.^20^

### 2.4 Data analysis

Statistical analysis was performed using SPSS 16.0 (SPSS, Inc) and GraphPad Prism (version 5.01, Inc). Data are reported as counts and percentages for categorical variables and means ± standard deviation (SD) or median and interquartile range for continuous variables. Based on the MMSE, patients were divided into two groups: with or without cognitive impairment on Day 30 postpoisoning. Between‐group comparisons were conducted using the chi‐square or Fisher's exact test for categorical variables and the Student's *t* test or the Mann‐Whitney *U* test for continuous variables. Univariate logistic regression analysis was used to select potential predictors of cognitive impairment. Variables with a *P* value <0.05 in univariate logistic regression were retained for multivariate logistic regression analysis. Those variables that attained a *P* value of 0.05 were considered significantly associated with cognitive impairment after adjustment for the other investigated covariates. Predictive values, estimations of optimal cutoff points, and area under the curve (AUC) were calculated from receiver operating characteristic (ROC) curves. All hypotheses were two‐tailed, and *P* < 0.05 was considered statistically significant.

## 3 RESULTS

### 3.1 Baseline characteristics of the study patients

Overall, 297 patients were eligible for this study, and 238 patients were included. Of these, 21 patients died, and ten were lost to follow‐up. Data from 207 patients (89 men [43.0%] and 118 women [57.0%]) with a mean age of 54.7 ± 18.3 years (range, 16 to 72 years) were included in the final analysis (Table 1). Among the 207 patients, 192 patients had intentionally and 15 patients had unintentionally consumed an organophosphorus pesticide. Median time from poisoning to treatment was 4.5 hours (IQR 2.2‐6.5 hours). A total of 207 patients were diagnosed with poisoning from an organophosphate compound, including dichlorvos (n = 52), rogor (n = 34), methamidophos (n = 22), omethoate (n = 20), thimet (n = 18), triazophos (n = 16), parathion (n = 16), malathion (n = 14), phoxim (n = 9), and unknown organophosphorus pesticide (n = 6). Median serum cholinesterase activity of the patients on admission to hospital (Day 1 postpoisoning) was 12.2% (range, 4.39%‐25.47%). Serum UCH‐L1 levels on admission were significantly higher in AOPP patients [5.13 (2.92‐7.54) ng/mL; n = 207] compared to healthy controls (0.27 ± 0.13 ng/mL; n = 102) (*P* < 0.001).

**Table 1:** Baseline characteristics of included patients

Variable	Cognitive impairment on Day 30		All (n = 207)	*P* value
	Without (n = 171)	With (n = 36)		
Age (y)	51.5 ± 20.4	56.2 ± 15.8	54.7 ± 18.3	0.322
Gender (M/F)	96/75	22/14	118/89	0.584
Time from poisoning to treatment (h)	4.8 (2.0‐6.9)	4.1 (1.6‐7.0)	4.5 (2.2‐6.5)	0.632
Body temperature (°C)	36.4 ± 0.7	37.0 ± 0.5	36.9 ± 0.6	0.173
Heart rate (beats/min)	81.4 ± 16.9	88.7 ± 22.6	85.3 ± 21.4	0.115
Respiratory rate (respirations/min)	16.4 ± 3.8	19.0 ± 4.9	18.2 ± 4.0	0.344
Mean arterial pressure (mm Hg)	90.2 ± 18.6	84.4 ± 26.2	87.3 ± 20.1	0.131
APACHE II score	18.03 ± 6.65	29.74 ± 7.08	24.32 ± 6.86	<0.001
Serum cholinesterase level (U/L)	680.5 (247.0‐1747.5)	325.0 (130.5‐725.0)	549.0 (197.5‐1146.0)	0.116
Serum cholinesterase activity (%)	15.12 (5.49‐38.83)	7.22 (2.9‐16.11)	12.2 (4.39‐25.47)	0.116
Lactate (mmol/L)	2.65 (0.44‐4.82)	4.35 (2.52‐6.23)	3.42 (1.37‐5.40)	0.010
pH	7.32 ± 0.18	7.27 ± 0.10	7.30 ± 0.15	0.142
${\text{HCO}}_{3}^{-}$level (mmol/L)	18.44 ± 5.47	17.53 ± 6.45	18.02 ± 5.97	0.437
White blood cell (×10^9^/L)	9.35 (3.73‐14.05)	14.96 (8.06‐19.32)	12.15 (6.35‐17.08)	0.008
C‐reactive protein level (mg/L)	20.80 ± 9.21	24.72 ± 7.84	22.57 ± 8.18	0.107
Serum UCH‐L1_Day1_ levels	4.25 (2.57‐6.31)	8.51 (6.53‐10.22)	5.13 (2.92‐7.54)	<0.001
Duration of ICU (h)	5.15 (3.10‐7.15)	5.75 (2.95‐8.60)	5.60 (2.60‐8.05)	0.709
Mechanical ventilation time (h)	3.55 (1.05‐6.10)	4.80 (0.85‐8.35)	4.05 (0.50‐7.25)	0.334

Note

Numerical variables are presented as mean ± standard deviation (SD) or median (interquartile range) and were analyzed using the unpaired Student's *t* test or Mann‐Whitney *U* test. Categorical variables are expressed as counts and were analyzed using the chi‐square test.

Abbreviations: APACHE II, Acute Physiology and Chronic Health Evaluation II; UCH‐L1, ubiquitin C‐terminal hydrolase L1.

### 3.2 Prediction of cognitive impairment

**Figure 1:**
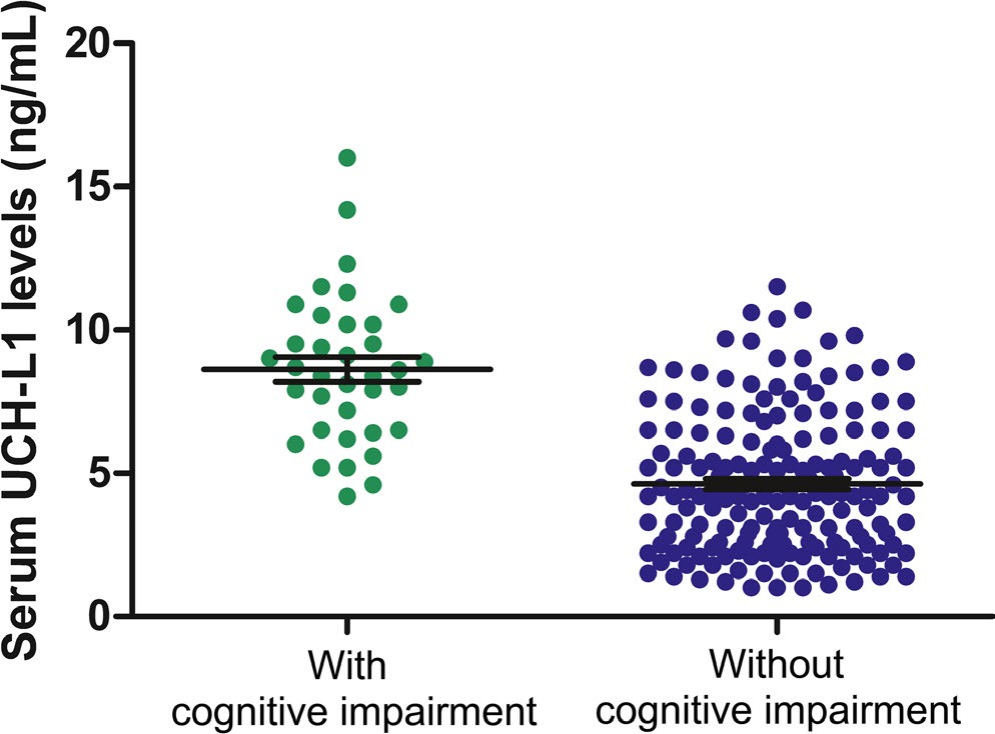
Serum UCH‐L1 levels on hospital admission (Day 1 postpoisoning) in patients with and without cognitive impairment on Day 30 postpoisoning. Serum UCH‐L1 levels on admission were significantly higher in patients with cognitive impairment [8.51 (6.53‐10.22) ng/mL; n = 36] compared to those without [4.25 (2.57‐6.31) ng/mL; n = 171] (*P* < 0.001). Black horizontal lines indicate the mean, and error bars indicate standard errors. Statistical analysis was performed using the Mann‐Whitney *U* test

**Figure 2:**
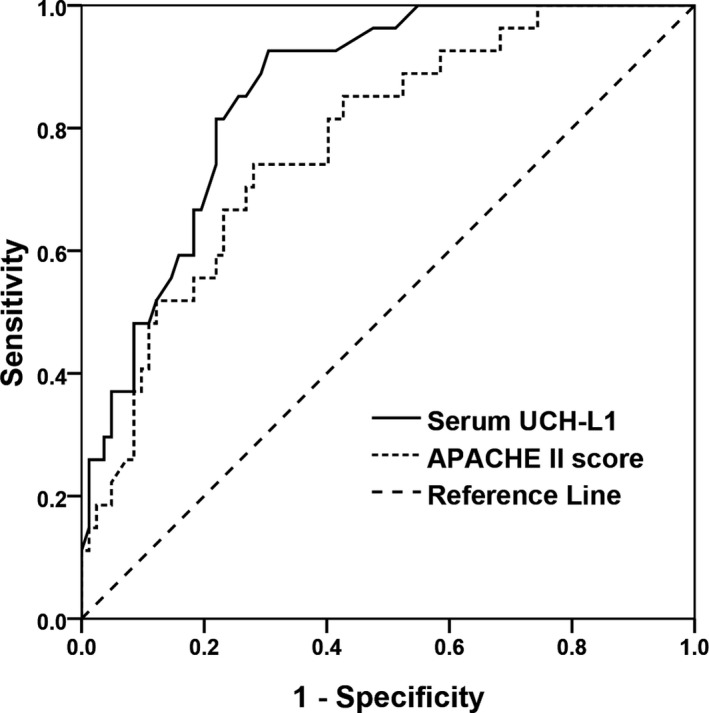
Receiver operating characteristic curve for serum UCH‐L1 levels, 24‐h APACHE II score and the development of cognitive impairment on Day 30 postpoisoning in patients with AOPP

Among the 207 patients with AOPP, 36 patients (17.4%) had cognitive impairment on Day 30 postpoisoning. Serum UCH‐L1 levels on admission to hospital (Day 1 postpoisoning) were significantly higher in patients with cognitive impairment [8.51 (6.53‐10.22) ng/mL; n = 36] compared to those without [4.25 (2.57‐6.31) ng/mL; n = 171] (*P* < 0.001) (Figure 1). 24‐h APACHE II score, blood lactate, and white blood cell count were also significantly higher in patients with AOPP and cognitive impairment (Table 1). On univariate analysis, serum UCH‐L1 levels on admission, 24‐h APACHE II score, blood lactate level, and white blood cell count were significantly associated with cognitive impairment on Day 30 postpoisoning (Table 2). On multivariate analysis, serum UCH‐L1 levels on admission (odds ratio [OR] 1.889, 95% confidence interval [CI] 1.609‐3.082, *P* = 0.002) and 24‐h APACHE II score (OR 1.736, 95% CI 1.264‐3.272, *P* = 0.012) were independent predictors of cognitive impairment on Day 30 postpoisoning. Based on ROC curves, serum UCH‐L1 levels > 5.9 ng/mL on admission predicted development of cognitive impairment on Day 30 postpoisoning with 86.1% sensitivity and 72.5% specificity (AUC, 0.869; 95% CI 0.815‐0.923), and a 24‐hr APACHE II score > 23 predicted development of cognitive impairment on Day 30 postpoisoning with 74.1% sensitivity and 68.3% specificity (AUC: 0.773, 95% CI: 0.681‐0.876). Serum UCH‐L1 level had better prognostic value than the 24‐h APACHE II score for predicting cognitive impairment on Day 30 postpoisoning vs. no cognitive impairment (Figure 2).

**Table 2:** Univariate and multivariate analysis of clinical variables for cognitive impairment at Day 30 postpoisoning

Variables	Univariate analysis		Multivariate analysis	
	HR (95% confidence interval)	*P* value	HR (95% confidence interval)	*P* value
APACHE II score	2.117 (1.682‐3.502)	<0.001	1.736 (1.264‐3.272)	0.012
Lactate	2.072 (1.426‐2.741)	0.024	1.883 (1.389‐2.671)	0.098
White blood cell	1.962 (1.334‐2.465)	0.018	1.708 (1.268‐2.245)	0.076
Serum UCH‐L1 level on Day 1 postpoisoning	2.312 (1.746‐3.804)	<0.001	1.889 (1.609‐3.082)	0.002

### 3.3 Time course of serum UCH‐L1 levels

**Figure 3:**
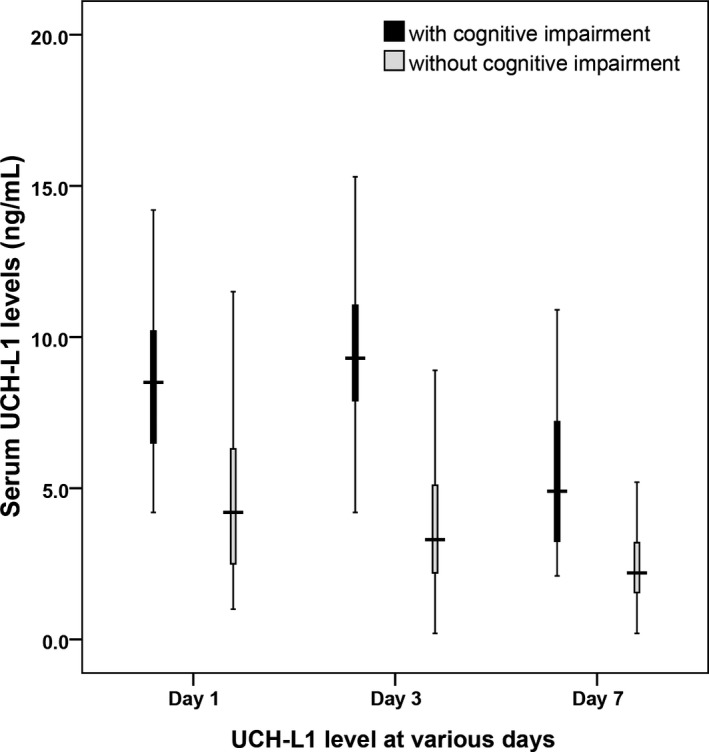
Serum UCH‐L1 concentrations on hospital admission (Day 1 postpoisoning) and Day 3 and Day 7 postpoisoning

In patients with cognitive impairment on Day 30 postpoisoning, serum UCH‐L1 concentration peaked on Day 3 postpoisoning and slowly decreased over time, while serum UCH‐L1 concentration decreased with time in patients without cognitive impairment (Figure 3). Serum UCH‐L1 concentration did not reach the normal range on Day 7 postpoisoning. On admission to hospital (Day 1 postpoisoning) [8.51 (6.53‐10.22) ng/mL vs 4.25 (2.57‐6.31) ng/mL, *P* < 0.001) and Day 3 [9.31 (7.92‐10.98) ng/mL vs 3.32 (2.25‐5.13) ng/mL, *P* < 0.001] and Day 7 [4.96 (3.28‐7.26) ng/mL vs 2.27 (1.55‐3.24) ng/mL, *P* < 0.001] postpoisoning, serum UCH‐L1 levels were significantly higher in patients with cognitive impairment compared to those without.

## 4 DISCUSSION

This study investigated the demographic and clinical parameters that are associated with cognitive impairment on Day 30 postpoisoning in patients with AOPP. Findings showed that serum UCH‐L1 levels on admission to hospital (Day 1 postpoisoning) were significantly higher in patients that presented with cognitive impairment on Day 30 postpoisoning compared to those that did not. Multivariate logistic regression analysis identified levels of serum UCH‐L1 on admission and 24‐h APACHE II score as independent predictors of cognitive impairment in patients with AOPP.

Organophosphate compounds induce cholinergic neuronal excitotoxicity, which can cause persistent profound neuropsychiatric and neurological impairments, including memory, cognitive, mental, emotional, motor, and sensory deficits. The underlying mechanisms involve cellular edema, oxidative stress, cytotoxicity, neuroinflammation, and neuronal apoptosis.^21^ Brain magnetic resonance imaging (MRI) findings in patients after organophosphate poisoning showed localized high signal intensity lesions in the white matter.^22^ An animal study associated organophosphate poisoning with reduced white matter integrity within the striatum and amygdala that correlated with spatial learning performance.^23^

UCH‐L1 is a soluble protein localized in the cell body of neurons in the central nervous system that has important roles in the regulation of synaptic plasticity and learning and memory.^24^ Circulating UCH‐L1 has been identified as a biomarker specific to neuronal injury^16^, ^25^ as it is released into the circulation when the integrity of the blood‐brain barrier (BBB) is disrupted.^26^, ^27^ In a piglet model, serum UCH‐L1 predicted neuronal apoptosis induced by deep hypothermic circulatory arrest.^28^ In observational studies, cerebrospinal fluid (CSF) and serum levels of UCH‐L1 had utility as diagnostic and prognostic biomarkers of traumatic brain injury.^13^, ^14^, ^29^ In patients with aneurysmal subarachnoid hemorrhage, elevated CSF levels of UCH‐L1 correlated with neurological outcomes and mortality.^15^ In patients with white matter lesions, increased serum UCH‐L1 levels were correlated with white matter lesion severity.^30^ Taken together, these data and findings from the present study suggest that serum UCH‐L1 level has potential as a predictive marker for cognitive impairment after AOPP. This study showed that serum UCH‐L1 levels >5.9 ng/mL could predict the development of cognitive impairment after AOPP with relatively high sensitivity and specificity. These results are consistent with our previous report describing the prognostic value of serum UCH‐L1 levels in acute carbon monoxide poisoning.^31^ Other studies suggest optimal UCH‐L1 values for predicting outcomes differ between disease types. In patients with traumatic brain injury, the UCH‐L1 cutoff value for the prediction of poor outcomes was 1.03 ng/mL.^29^ In patients with severe traumatic brain injury, the UCH‐L1 cutoff value for in‐hospital mortality was 1.89 ng/mL.^32^

APACHE II scoring is a classification system that is used to evaluate the severity and prognosis of disease.^33^ In the present study, APACHE II score was significantly higher in patients with AOPP that presented with cognitive impairment on Day 30 postpoisoning compared to those that did not. Accordingly, a previous report showed that APACHE II score had the capability to discriminate and estimate early in‐hospital mortality in patients with AOPP.^34^ However, the APACHE II score is highly complex as it evaluates disease severity based on 12 physiological measurements taken at admission, patient age, and patient medical history. Consequently, the APACHE II score may not be easily applied in the Emergency Department.

Published evidence suggests that blood lactate and leukocyte level can be used as markers of severity and prognosis in patients with AOPP.^35^, ^36^ In the present study, blood lactate level and white blood cell count were significantly higher in patients with AOPP that presented with cognitive impairment on Day 30 postpoisoning compared to those that did not. However, blood lactate level and white blood cell count were not selected as independent predictive factors for cognitive impairment on multivariate analysis. An evidence‐based review of the management of AOPP recommends quantification of erythrocyte AchE activity during the initial diagnosis of AOPP. Erythrocyte and synaptic AchE are structurally similar; therefore, erythrocyte AchE activity is thought to reflect synaptic AchE activity.^37^ Plasma cholinesterase activity has also been accepted as a biomarker of exposure/toxicity in AOPP; however, more recent reports suggest that plasma cholinesterase activity does not accurately reflect the severity of AOPP as it is not involved in cholinergic transmission in the nervous system.^4^ Accordingly, the present study found no significant differences in serum cholinesterase activity in patients that presented with cognitive impairment on Day 30 postpoisoning compared to those that did not.

The present study was associated with several limitations. First, the sample size was small. Second, the study was conducted in a single hospital and may not be generalizable to other healthcare settings or patient populations. Third, the incidence of cognitive impairment may have been underestimated as the follow‐up period of 30 days was relatively short. Last, assessment of erythrocyte AchE would have provided valuable information; however, erythrocyte AchE cannot be measured at our institution. Further studies with larger sample sizes are warranted to validate our findings.

## 5 CONCLUSION

In conclusion, this study demonstrates that serum UCH‐L1 level measured at the time of hospital admission has potential as a novel biomarker for predicting cognitive impairment 30 days after AOPP.

## References

[1] Hrabetz H , Thiermann H , Felgenhauer N , et al. Organophosphate poisoning in the developed world ‐ A single centre experience from here to the millennium. Chem Biol Interact. 2013;206:561‐568. 23685200 10.1016/j.cbi.2013.05.003

[2] Terry AV Jr . Functional consequences of repeated organophosphate exposure: Potential non‐cholinergic mechanisms. Pharmacol Ther. 2012;134:355‐365. 22465060 10.1016/j.pharmthera.2012.03.001 PMC3366364

[3] Malekirad AA , Faghih M , Mirabdollah M , Kiani M , Fathi A , Abdollahi M . Neurocognitive, mental health, and glucose disorders in farmers exposed to organophosphorus pesticides. Arh Hig Rada Toksikol. 2013;64:1‐8. 23705196 10.2478/10004-1254-64-2013-2296

[4] Jokanovic M . Neurotoxic effects of organophosphorus pesticides and possible association with neurodegenerative diseases in man: A review. Toxicology. 2018;410:125‐131. 30266654 10.1016/j.tox.2018.09.009

[5] Pancetti F , Olmos C , Dagnino‐Subiabre A , Rozas C , Morales B . Noncholinesterase effects induced by organophosphate pesticides and their relationship to cognitive processes: Implication for the action of acylpeptide hydrolase. J Toxicol Environ Health B Crit Rev. 2007;10:623‐630. 18049927 10.1080/10937400701436445

[6] Bishop P , Rocca D , Henley JM . Ubiquitin C‐terminal hydrolase L1 (UCH‐L1): structure, distribution and roles in brain function and dysfunction. Biochem J. 2016;473:2453‐2462. 27515257 10.1042/BCJ20160082 PMC4980807

[7] Hegde AN , DiAntonio A . Ubiquitin and the synapse. Nat Rev Neurosci. 2002;3:854‐861. 12415293 10.1038/nrn961

[8] Bourtchouladze R , Lidge R , Catapano R , et al. A mouse model of Rubinstein‐Taybi syndrome: Defective long‐term memory is ameliorated by inhibitors of phosphodiesterase 4. Proc Natl Acad Sci USA. 2003;100:10518‐10522. 12930888 10.1073/pnas.1834280100 PMC193593

[9] Contu VR , Kotake Y , Toyama T , et al. Endogenous neurotoxic dopamine derivative covalently binds to Parkinson's disease‐associated ubiquitin C‐terminal hydrolase L1 and alters its structure and function. J Neurochem. 2014;130:826‐838. 24832624 10.1111/jnc.12762

[10] Jara JH , Genç B , Cox GA , et al. Corticospinal Motor Neurons Are Susceptible to Increased ER Stress and Display Profound Degeneration in the Absence of UCHL1 Function. Cereb Cortex. 2015;25:4259‐4272. 25596590 10.1093/cercor/bhu318 PMC4626833

[11] Naze P , Vuillaume I , Destee A , Pasquier F , Sablonniere B . Mutation analysis and association studies of the ubiquitin carboxyterminal hydrolase L1 gene in Huntington's disease. Neurosci Lett. 2002;328:1‐4. 12123845 10.1016/s0304-3940(02)00231-8

[12] Li Y , Wang Z , Zhang B , et al. Cerebrospinal fluid ubiquitin C‐terminal hydrolase as a novel marker of neuronal damage after epileptic seizure. Epilepsy Res. 2013;103:205‐210. 22920679 10.1016/j.eplepsyres.2012.08.001

[13] Diaz‐Arrastia R , Wang K , Papa L , et al. Acute Biomarkers of Traumatic Brain Injury: Relationship between Plasma Levels of Ubiquitin C‐Terminal Hydrolase‐L1 and Glial Fibrillary Acidic Protein. J Neurotrauma. 2014;31:19‐25. 23865516 10.1089/neu.2013.3040 PMC3880090

[14] Bazarian JJ , Biberthaler P , Welch RD , et al. Serum GFAP and UCH‐L1 for prediction of absence of intracranial injuries on head CT (ALERT‐TBI): a multicentre observational study. Lancet Neurol. 2018;17:782‐789. 30054151 10.1016/S1474-4422(18)30231-X

[15] Lewis SB , Wolper R , Chi Y‐Y , et al. Identification and preliminary characterization of ubiquitin c terminal hydrolase 1 (UCHL1) as a biomarker of neuronal loss in aneurysmal subarachnoid hemorrhage. J Neurosci Res. 2010;88:1475‐1484. 20077430 10.1002/jnr.22323

[16] Ren C , Kobeissy F , Alawieh A , et al. Assessment of serum UCH‐L1 and GFAP in acute stroke patients. Sci Rep. 2016;6(1). 10.1038/srep24588 PMC4830936 27074724

[17] Douglas‐Escobar MV , Heaton SC , Bennett J , et al. UCH‐L1 and GFAP serum levels in neonates with hypoxic‐ischemic encephalopathy: a single center pilot study. Frontiers in Neurology. 2014;5. 10.3389/fneur.2014.00273 PMC4271579 25566179

[18] Fink EL , Berger RP , Clark R , et al. Exploratory study of serum ubiquitin carboxyl‐terminal esterase L1 and glial fibrillary acidic protein for outcome prognostication after pediatric cardiac arrest. Resuscitation. 2016;101:65‐70. 26855294 10.1016/j.resuscitation.2016.01.024 PMC4792689

[19] Brophy GM , Mondello S , Papa L , et al. Biokinetic analysis of ubiquitin C‐terminal hydrolase‐L1 (UCH‐L1) in severe traumatic brain injury patient biofluids. J Neurotrauma. 2011;28:861‐870. 21309726 10.1089/neu.2010.1564 PMC3113451

[20] Folstein MF , Folstein SE , McHugh PR . Mini‐mental state ‐ practical method for grading cognitive state of patients for clinician. J Psychiatr Res. 1975;12:189‐198. 1202204 10.1016/0022-3956(75)90026-6

[21] Chen Y . Organophosphate‐induced brain damage: Mechanisms, neuropsychiatric and neurological consequences, and potential therapeutic strategies. Neurotoxicology. 2012;33:391‐400. 22498093 10.1016/j.neuro.2012.03.011

[22] Lee K‐J , Shin J‐W , Moon J , et al. An illustrative case of mixed pesticide poisoning with remarkable improvement: A case report. J Neurol Sci. 2014;344:232‐233. 24993470 10.1016/j.jns.2014.06.027

[23] Mullins RJ , Xu SU , Pereira E , et al. Prenatal exposure of guinea pigs to the organophosphorus pesticide chlorpyrifos disrupts the structural and functional integrity of the brain. Neurotoxicology. 2015;48:9‐20. 25704171 10.1016/j.neuro.2015.02.002 PMC4442734

[24] Guo Y‐Y , Lu YI , Zheng Y , et al. Ubiquitin C‐terminal hydrolase L1 (UCH‐L1) promotes hippocampus‐dependent memory via its deubiquitinating effect on TrkB. J Neurosci. 2017;37:5978‐5995. 28500221 10.1523/JNEUROSCI.3148-16.2017 PMC6596500

[25] Mondello S , Shear DA , Bramlett HM , et al. insight into pre‐clinical models of traumatic brain injury using circulating brain damage biomarkers: operation brain trauma therapy. J Neurotrauma. 2016;33:595‐605. 26671651 10.1089/neu.2015.4132

[26] Carone D , Librizzi L , Cattalini A , et al. Pravastatin acute neuroprotective effects depend on blood brain barrier integrity in experimental cerebral ischemia. Brain Res. 2015;1615:31‐41. 25912435 10.1016/j.brainres.2015.04.025

[27] Zhang D , Han S , Wang S , Luo Y , Zhao L , Li J . cPKC gamma‐mediated down‐regulation of UCHL1 alleviates ischaemic neuronal injuries by decreasing autophagy via ERK‐mTOR pathway. J Cell Mol Med. 2017;21:3641‐3657. 28726275 10.1111/jcmm.13275 PMC5706506

[28] Zhang Y‐P , Zhu Y‐B , Duan DD , et al. Serum UCH‐L1 as a Novel Biomarker to Predict Neuronal Apoptosis Following Deep Hypothermic Circulatory Arrest. Int J Med Sci. 2015;12:576‐582. 26180514 10.7150/ijms.12111 PMC4502062

[29] Takala R , Posti JP , Runtti H , et al. Glial Fibrillary Acidic Protein and Ubiquitin C‐Terminal Hydrolase‐L1 as Outcome Predictors in Traumatic Brain Injury. World Neurosurg. 2016;87:8‐20. 26547005 10.1016/j.wneu.2015.10.066

[30] Li Y , Sun Y , Li J , et al. Changes of ubiquitin C‐terminal hydrolase‐L1 levels in serum and urine of patients with white matter lesions. J Neurol Sci. 2015;357:215‐221. 26232084 10.1016/j.jns.2015.07.033

[31] Pang LI , Wu Y , Dong N , et al. Elevated serum ubiquitin C‐terminal hydrolase‐L1 levels in patients with carbon monoxide poisoning. Clin Biochem. 2014;47:72‐76. 24080464 10.1016/j.clinbiochem.2013.09.015

[32] Mondello S , Papa L , Buki A , et al. Neuronal and glial markers are differently associated with computed tomography findings and outcome in patients with severe traumatic brain injury: a case control study. Crit Care. 2011;15:R156. 21702960 10.1186/cc10286 PMC3219030

[33] Akdur O , Durukan P , Ozkan S , et al. Poisoning severity score, Glasgow coma scale, corrected QT interval in acute organophosphate poisoning. Human Exper Toxicol. 2010;29:419‐425. 10.1177/0960327110364640 20203133

[34] Kim YH , Yeo JH , Kang MJ , et al. Performance Assessment of the SOFA, APACHE II Scoring System, and SAPS II in Intensive Care Unit Organophosphate Poisoned Patients. J Korean Med Sci. 2013;28:1822‐1826. 24339715 10.3346/jkms.2013.28.12.1822 PMC3857381

[35] Yuan S , Gao Y , Ji W , Song J , Mei X . The evaluation of acute physiology and chronic health evaluation II score, poisoning severity score, sequential organ failure assessment score combine with lactate to assess the prognosis of the patients with acute organophosphate pesticide poisoning. Medicine. 2018;97. 10.1097/MD.0000000000010862 PMC6392888 29794787

[36] Kumar S , Agrawal S , Raisinghani N , Khan S . Leukocyte count: A reliable marker for the severity of organophosphate intoxication? J Lab Physicians. 2018;10:185‐188. 29692585 10.4103/JLP.JLP_100_17 PMC5896186

[37] Hmoudo H , Ben Salem C , Bouraoui K . Management of acute organophosphorus pesticide poisoning. Lancet. 2008;371:2169‐2170. 10.1016/S0140-6736(08)60946-0 18586164

